# Characterization of Type I Interferon-Associated Chemokines and Cytokines in Lacrimal Glands of Nonobese Diabetic Mice

**DOI:** 10.3390/ijms22073767

**Published:** 2021-04-05

**Authors:** Merri-Grace Allred, Michael S. Chimenti, Ashley E. Ciecko, Yi-Guang Chen, Scott M. Lieberman

**Affiliations:** 1 Stead Family Department of Pediatrics, Carver College of Medicine, University of Iowa, Iowa City, IA 52242, USA; merri-grace-allred@uiowa.edu; 2 Immunology Graduate Program, University of Iowa, Iowa City, IA 52242, USA; 3 Iowa Institute of Human Genetics, Carver College of Medicine, University of Iowa, Iowa City, IA 52242, USA; michael-chimenti@uiowa.edu; 4 Department of Pediatrics, Medical College of Wisconsin, Milwaukee, WI 53226, USA; aciecko@mcw.edu (A.E.C.); yichen@mcw.edu (Y.-G.C.); 5 Department of Microbiology and Immunology, Medical College of Wisconsin, Milwaukee, WI 53226, USA; 6 Max McGee National Research Center for Juvenile Diabetes, Medical College of Wisconsin, Milwaukee, WI 53226, USA

**Keywords:** Sjögren’s disease, lacrimal gland, nonobese diabetic mice, type I interferon, interleukin-21, chemokines

## Abstract

Type I interferons (IFNs) are required for spontaneous lacrimal gland inflammation in the nonobese diabetic (NOD) mouse model of Sjögren’s disease, but the consequences of type I IFN signaling are not well-defined. Here, we use RNA sequencing to define cytokine and chemokine genes upregulated in lacrimal glands of NOD mice in a type I IFN-dependent manner. Interleukin (IL)-21 was the highest differentially expressed cytokine gene, and *Il21* knockout NOD mice were relatively protected from lacrimal gland inflammation. We defined a set of chemokines upregulated early in disease including *Cxcl9* and *Cxcl10*, which share a receptor, CXCR3. CXCR3^+^ T cells were enriched in lacrimal glands with a dominant proportion of CXCR3^+^ regulatory T cells. Together these data define the early cytokine and chemokine signals associated with type I IFN-signaling in the development of lacrimal gland inflammation in NOD mice providing insight into the role of type I IFN in autoimmunity development.

## 1. Introduction

Sjögren’s disease (SD) is a chronic autoimmune disease that targets the lacrimal and salivary glands. Early pathogenesis is characterized by the infiltration of lymphocytes in these tissues leading to foci of inflammation and glandular destruction. In later stages of the disease, the autoimmune response leads to reduced production of tears and saliva resulting in progressive dry eyes and mouth and associated complications including poor oral health and vision-threatening ocular surface damage. Extra-glandular manifestations may occur targeting nearly any organ, and individuals with SD often experience significant pain and fatigue. Individuals with SD are also at risk for lymphoma, pregnancy complications such as neonatal lupus, and overall decreased quality of life. Treatments may provide some improvement in symptoms, but no treatments have demonstrated consistent reversal of clinical manifestations.

Type I interferons (IFNs) are cytokines that have broad effects on the immune system including stimulation and modulation of both innate and adaptive immune cells to drive inflammatory responses. While the IFN system is believed to have developed as a key anti-viral mechanism, the role of IFNs in multiple autoimmune diseases, including SD, has been demonstrated [1,2,3,4]. IFNs drive both innate and adaptive immune cell activities including T and B cell proliferation, macrophage activation and NK cell function [5]. In SD, IFN-stimulated genes are upregulated in the minor salivary glands, ocular epithelial cells, peripheral mononuclear cells, monocytes and B cells [6,7,8,9,10,11]. Both type I and type II IFNs have been implicated in the pathogenesis of SD. Gene expression studies of target tissue from humans with SD have demonstrated heterogeneity in the expression of the IFN signature. Labial minor salivary gland biopsy specimens from individuals with SD demonstrated an IFN signature in 58% of individuals, with further analyses identifying three IFN-related patterns nearly evenly distributed: patients with type I IFN dominant signature, those with type II IFN dominant signature, and those with a combined type I and type II IFN signature [12,13,14]. Whether these subsets represent multiple different pathways driving the early immune attack on salivary and lacrimal glands in the context of SD or, rather, different temporal stages of the inflammatory response is not known. 

Nonobese diabetic (NOD) mice develop spontaneous SD-like autoimmunity including similar immunohistopathology of lacrimal and salivary glands as observed in humans [15,16]. However, NOD mice develop lacrimal and salivary gland disease independently in a sex-dependent manner with males spontaneously developing lacrimal gland inflammation and females spontaneously developing salivary gland inflammation [17,18]. Prior studies have demonstrated a pathogenic role for IFN signaling in the development of SD-like manifestations in NOD mice with clear distinctions in IFN requirements based on sex and gland affected. Lacrimal gland disease (spontaneous in males) required type I IFN signaling [19], while salivary gland disease (spontaneous in females) required type II IFN signaling [20]. Thus, spontaneous, sex-based, organ-specific, SD-like autoimmunity in NOD mice models two of the IFN-dependencies observed in humans with lacrimal gland disease representing the type I IFN-dominant disease and salivary gland disease representing the type II IFN-dominant disease [19,20]. This provides a unique tool to define immunopathogenic mechanisms of disease development that may then be translated to human SD. 

In this study, we have used type I IFN signaling-deficient NOD mice to study the role of type I IFN signaling in lacrimal gland autoimmunity. Through lacrimal gland tissue RNA sequencing studies, we identified type I IFN-dependent immune pathways associated with lacrimal gland inflammation largely dominated by cytokines and chemokines. The top cytokine upregulated in a type I IFN-dependent manner was interleukin (IL)-21. New *Il21* knockout (KO) NOD mice were created and demonstrated significant protection in the development of lacrimal gland inflammation. Additional gene expression and flow cytometry studies implicate multiple chemokines including ligands for CXCR3 and enrichment of CXCR3-expressing T cells within inflamed lacrimal glands. Ultimately, defining the role of type I IFN signaling in the NOD mouse model of SD will provide insight into the early type I IFN-dependent pathogenic mechanisms in the development of lacrimal gland autoimmunity.

## 2. Results

### 2.1. Type I IFN Signaling Is Required for Lacrimal Gland Inflammation in NOD Mice 

**Figure 1:**
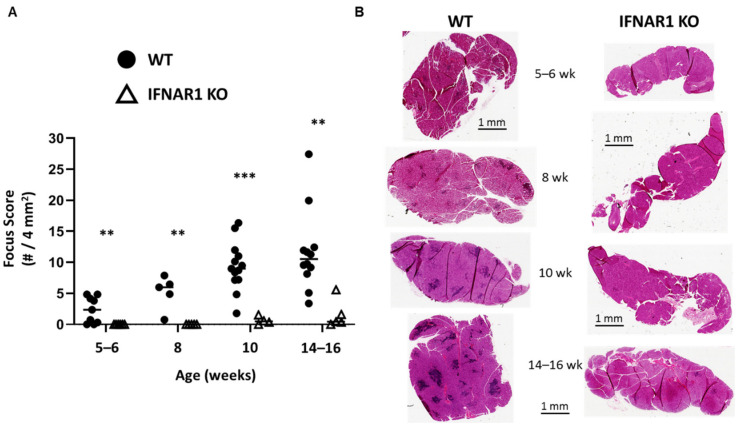
Type I IFN signaling is required for lacrimal gland inflammation. (**A**) Quantitation of lacrimal gland inflammation in WT or IFNAR1 KO NOD mice at different ages: 5-6 weeks (*n* = 6 KO, *n* = 9 WT), 8 weeks (*n* = 5 each), 10 weeks (*n* = 4 KO, *n* = 13 WT), 14–16 weeks (*n* = 5 KO, *n* = 12 WT) as indicated (x-axis). A focus is defined as an aggregate of ≥50 mononuclear cells, and focus score is equal to # of foci per 4 mm^2^ tissue. Symbols represent individual mice in each group (WT, filled circles; IFNAR1 KO, open triangles), lines are medians. ** *p* < 0.01, *** *p* < 0.001 by Mann-Whitney test. (**B**) Representative hematoxylin and eosin (H&E)-stained sections of lacrimal glands from WT or IFNAR1 KO NOD mice at indicated ages. Scale bars are 1 mm.

As we have shown previously, IFNAR1-deficient (IFNAR1 KO) NOD mice are protected from developing lacrimal gland inflammation. IFNAR1 is expressed by many immune and non-immune cells, and type I IFN signaling has many downstream effects [5,21]. To characterize the impact of type I IFN signaling on disease over time, lacrimal glands were harvested from wild-type (WT) and IFNAR1 KO male NOD mice at various time points. Our previous work demonstrated that by 14–16 weeks of age, WT male NOD mice develop robust inflammation compared to IFNAR1 KO NOD mice [19]. Here, we found lacrimal gland inflammation in some WT mice as early as five to six weeks of age with all WT mice developing some degree of focal infiltrates at eight weeks and increased inflammation over time (Figure 1). In contrast, no inflammation was detected in lacrimal glands of IFNAR1 KO mice before 10 weeks, and even then the inflammation was minimal (Figure 1). Even at 14–16 weeks, little inflammation was detected in IFNAR1 KO lacrimal glands except for one outlier (Figure 1). This suggests that type I IFN signaling is required for the early signals that disrupt immunological tolerance to promote lymphocytic infiltration of lacrimal glands in NOD mice.

### 2.2. RNA Sequencing of Whole Lacrimal Gland Implicates Key Innate and Adaptive Immune Pathways

**Figure 2:**
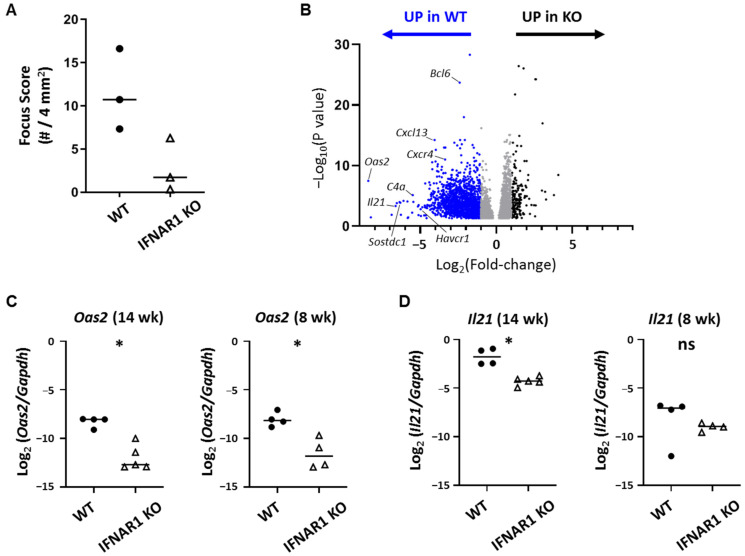
Type I IFN-dependent differential gene expression in lacrimal glands by RNA sequencing. (**A**) Quantitation of inflammation from WT and IFNAR1 KO NOD mouse lacrimal glands used for RNA sequencing studies. Focus scoring as described in Figure 1 legend. Symbols are individual mice, lines are medians. *p* = 0.1 by Mann-Whitney test. (**B**) Volcano plot of DE genes in lacrimal glands of NOD mice from (A) with genes up-regulated in WT or IFNAR1 KO as indicated above the graph. Dots represent individual genes with blue indicating at least 2-fold up-regulation in WT and black indicating at least 2-fold up-regulation in IFNAR1 KO. Gray genes are up-regulated with fold-change less than 2. Select genes up-regulated in WT are indicated. (C-D) Quantitation of *Oas2* (**C**) or *Il21* (**D**) gene expression in lacrimal glands of 14- or 8-week-old WT or IFNAR1 KO NOD mice (*n* = 4–5 per group) by qPCR. Graphs show log-transformed ratios of each gene normalized to *Gapdh*. Symbols represent individual mice, lines are medians. *p*-values by Mann-Whitney test, * *p* < 0.05, ^ns^ *p* > 0.05.

Given the many cell types that express IFNAR1, we performed whole tissue RNA sequencing studies to identify immune genes and pathways upregulated in the context of lacrimal gland autoimmunity in WT NOD mice compared to IFNAR1 KO NOD mice. For these studies, we isolated RNA from 20–21-week-old WT and IFNAR1 KO NOD mice. Samples from the IFNAR1 KO group had lower focus scores than those in the WT group (Figure 2A). Gene expression analyses identified 4371 significantly differentially expressed (DE) genes including 1854 genes with at least two-fold change between WT and IFNAR1 KO. Of these, 1663 were upregulated in WT compared to IFNAR1 KO lacrimal glands and 191 were upregulated in IFNAR1 KO glands (Figure 2B, Table 1, Supplemental Table S1). The top hit for DE genes upregulated in WT compared to IFNAR1 KO lacrimal glands was *Oas2*, which was expressed >340-fold higher in WT lacrimal glands (Table 1). We validated differential expression of *Oas2* with qPCR demonstrating upregulation of *Oas2* in WT lacrimal glands of 14-week-old NOD mice (Figure 2C). The upregulation of *Oas2* was also evident in WT lacrimal glands in 8-week-old NOD mice (Figure 2C), an age when inflammation was just becoming established (Figure 1), suggesting that downstream effects of type I IFN signaling were already apparent at this early time. Additional members of the OAS family were identified in our RNA sequencing analysis as DE with increased expression in WT lacrimal glands with 11–13-fold upregulation (*Oas3*, *Oasl2*), five to seven-fold upregulation (*Oas1g*, *Oas1a*), or ~four-fold upregulation (Oas1b, Oasl1, Oas1c) in WT lacrimal glands (Supplemental Table S1). Moreover, among those genes most highly DE in WT lacrimal glands was *Il21* (~100-fold) (Table 1), which we validated with qPCR as upregulated in 14-week-old NOD mice (Figure 2D). In contrast to *Oas2*, *Il21* was not uniformly upregulated in all lacrimal glands of younger (eight-week-old) WT NOD mice (Figure 2D). The genes we previously identified by qPCR as upregulated in lacrimal glands of WT compared to 14–16-week-old IFNAR1 KO NOD mice [19] were found to be upregulated in WT glands in these RNA sequencing studies including *Ccl19* (14-fold), *Cxcl9* (12.6-fold), *Ubd* (10.9-fold), and *Epsti1* (7.5-fold) (Table 1, Supplemental Table S1). 

Using iPathwayGuide to evaluate for processes and pathways overrepresented by DE gene enrichment, DE genes upregulated in WT lacrimal glands were enriched for biological processes associated with immune responses including both innate and adaptive immune responses (Table 2, Supplemental Table S2). Similarly, pathway analyses identified both innate and adaptive immune pathways with significant enrichment or overrepresentation (Table 3, Supplemental Table S3). 

### 2.3. Il21 KO NOD Mice Are Relatively Protected from Lacrimal Gland Inflammation

**Figure 3:**
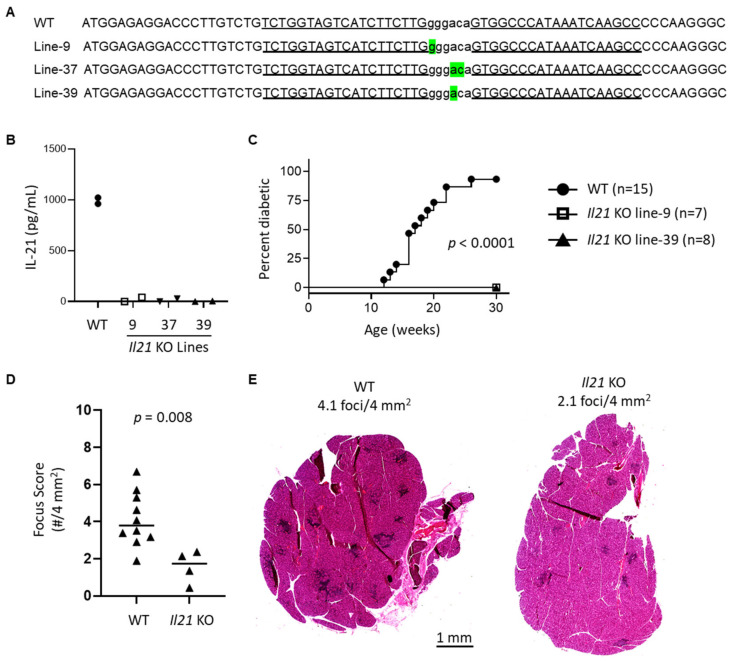
Generation and characterization of new *Il21* KO NOD strains. (**A**) ZFN-mediated mutagenesis of the *Il21* gene. The partial exon 1 sequence of the WT NOD *Il21* is shown at the top. The deleted nucleotides in *Il21* mutant lines were determined by DNA sequencing and are highlighted in green below the WT sequence. The ZFN target site is shown in lowercase letters and each of the ZFN binding sequences on the opposite strands is underlined. (**B**) *Il21* KO NOD mice do not produce IL-21. Splenocytes isolated from indicated strains were stimulated with soluble anti-CD3, and anti-CD28 in the presence of recombinant IL-6 for 3 days. Tissue culture media was collected and analyzed for IL-21 by ELISA in triplicate. Symbols represent mean of triplicate values for an individual mouse of the indicated genotype/line and are pooled from two independent experiments (1 mouse per group per experiment). (**C**) *Il21* KO NOD mice did not develop type 1 diabetes (T1D). Female mice were monitored weekly for T1D development. Diabetes onset was determined by two consecutive positive readings of glycosuria on a urine test strip. (**D**) Quantitation of lacrimal gland inflammation in WT (*n* = 10) and *Il21* KO (*n* = 4, including 1–2 from each KO line). Symbols, lines, and *p*-value as in Figure 1. (**E**) Representative H&E stained sections of lacrimal glands in (**D**) from WT or *Il21* KO NOD mice as indicated. Scale bar = 1 mm.

Given the previous association of IL-21 with SD [22] and our findings of *Il21* as a highly DE gene in WT lacrimal glands in older but not younger NOD mice, we wondered if IL-21 was required for lacrimal gland inflammation in NOD mice. To evaluate this, we created new lines of *Il21* KO NOD mice using zinc-finger nuclease (ZFN)-mediated mutagenesis directly in NOD embryos. One or two base pair deletions in the *Il21* gene were detected in three different lines (Figure 3A). IL-21 protein was not detected in stimulated splenocytes from any of these three lines (Figure 3B). Two of the lines were monitored for diabetes development and proved to be completely protected from the development of diabetes as expected based on prior studies of NOD mice deficient in IL-21 signaling [23,24,25] (Figure 3C). To evaluate the role of IL-21 in the development of lacrimal gland inflammation, we quantified inflammation in colony-matched, 10-week-old WT and *Il21* KO NOD mice and found a significant decrease in inflammation in mice lacking IL-21 (Figure 3D–E). Together these data demonstrate that IL-21 plays a pathogenic role in development of lacrimal gland inflammation in NOD mice. 

### 2.4. Characterization of Chemokine Expression in Lacrimal Glands of NOD Mice

**Figure 4:**
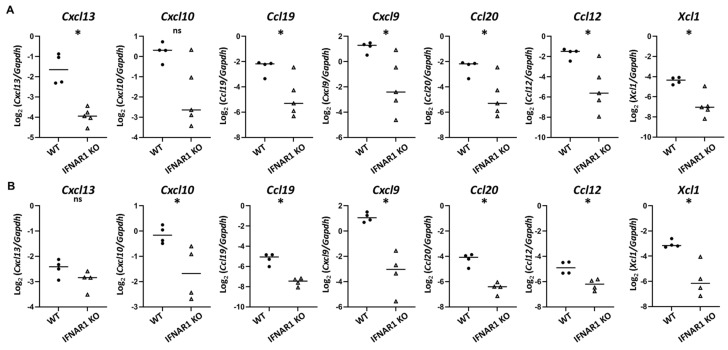
Identification of type I IFN-dependent chemokines upregulated in lacrimal glands of young NOD mice. (**A**,**B**) Graphs depict gene expression of indicated genes by qPCR from lacrimal glands of 14-week-old (**A**) or 8-week-old (**B**) WT or IFNAR1 KO NOD mice (*n* = 4-5 mice per group). Symbols represent individual mice, lines are medians. *p*-values by Mann-Whitney test with * *p* < 0.05, ^ns^ *p* > 0.05.

While IL-21 is clearly a disease-relevant cytokine downstream of IFNAR1-signaling, the lack of a significant difference in *Il21* expression in young NOD mice (Figure 2D) suggested that additional type I IFN-dependent signals were likely involved in early lacrimal gland inflammation. The two most highly overrepresented pathways identified in the RNA sequencing studies were cytokine-cytokine receptor interaction pathway and viral protein interactions with cytokine and cytokine receptor pathway (Table 3, Table 4 and Table 5, Supplemental Tables S3–S5). Given the role of chemokines in directing immune cell migration to, infiltration of, and localization within target tissues in the context of an immune response, we considered the differential expression of chemokines further through qPCR analyses. Specifically, we considered chemokines identified by RNA sequencing as being upregulated 12–16-fold (*Cxcl13*, *Cxcl10*, *Ccl19*, *Cxcl9*, *Ccl20*) or 6–10-fold (*Ccl12*, *Xcl1*) in WT lacrimal glands (Table 4 and Table 5). Similar to our prior findings of *Cxcl9* and *Ccl19* upregulation in 14–16-week-old WT compared to IFNAR1 KO lacrimal glands [19], qPCR of lacrimal glands from 14-week-old NOD mice demonstrated increased expression of these chemokines compared to IFNAR1 KO lacrimal glands (Figure 4A). In addition, at 14-weeks, *Cxcl13*, *Ccl20*, *Ccl12*, and *Xcl1* were each expressed at significantly higher levels in lacrimal glands from WT NOD mice compared to those of IFNAR1 KO NOD mice (Figure 4A). To determine which chemokines were upregulated at an earlier time in disease development, we evaluated gene expression in 8-week-old NOD mice and found that even at this early time point *Cxcl10*, *Cxcl9*, *Ccl12*, *Ccl20*, *Ccl19*, and *Xcl1* were each expressed at significantly higher levels in WT lacrimal glands, while *Cxcl13* was not (Figure 4B). 

### 2.5. CXCR3-Expressing T Cells Are Abundant in Inflamed Lacrimal Glands

**Figure 5:**
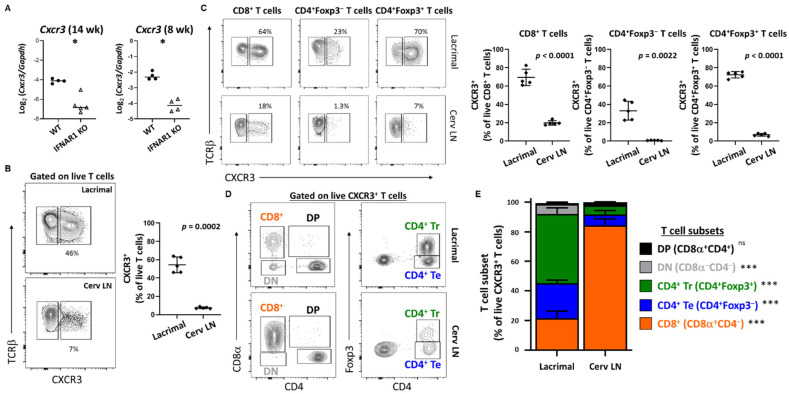
CXCR3^+^ T cells are enriched in lacrimal glands of WT NOD mice. (**A**) Quantitation of *Cxcr3* in lacrimal glands of 14-week-old (left) and 8-week-old (right) WT and IFNAR1 KO NOD mice, shown as Log-transformed ratio normalized to *Gapdh*. Symbols represent individual mice (*n* = 4–5 per group), lines are medians. *p*-values by Mann-Whitney test with * *p* < 0.05. (**B**) Representative contour plots gated on live T cells (TCRβ^+^CD19^−^ singlets) isolated from lacrimal glands (top) or cervical lymph nodes (Cerv LN, bottom) of WT NOD mice. Numbers indicate % CXCR3^+^ cells. The graph (right) depicts the cumulative proportion of CXCR3^+^ cells among live T cells in lacrimal glands or cerv LNs from WT NOD mice (*n* = 5). Symbols represent individual mice, lines are means, and error bars are standard deviation. *p*-value by paired t-test. (**C**) Representative contour plots demonstrate CXCR3^+^ populations of live T cell (TCRβ^+^CD19^−^) subsets gated as indicated above graphs, isolated from lacrimal (top) or cerv LN (bottom). Graphs depict cumulative data represented in the contour plots from multiple WT NOD mice (*n* = 5). Symbols, lines, error bars, and *p*-values as in (B). (**D**) Representative contour plots gated on live CXCR3^+^ T cells (TCRβ^+^CD19^−^) showing the different T cell subsets further quantified in (**E**). (**E**) Stacked bar graphs demonstrating the proportions of different T cell subsets represented in (**D**) that make up the CXCR3^+^ T cell population represented in (**B**). Each stacked bar represents the mean % of CXCR3^+^ cells within the indicated T cell subset with error bars representing standard deviation (*n* = 5 mice). *p*-values for comparisons of each subset between lacrimal and cerv LN by paired t-test indicated in key with *** *p* < 0.001, ^ns^ *p* > 0.05. DN, double negative; DP, double positive; Te, effector T cells; Tr, regulatory T cells.

Two of the chemokines identified as being upregulated in WT lacrimal glands at an early stage in lacrimal gland inflammation (i.e., CXCL9 and CXCL10) share a receptor–CXCR3. *Cxcr3* was significantly DE with over seven-fold increase in WT compared to IFNAR1 KO lacrimal glands in the RNA sequencing studies (Table 5, Supplemental Table S1). By qPCR, we found *Cxcr3* upregulated in lacrimal glands of 14-week-old WT compared to IFNAR1 KO NOD mice and in lacrimal glands of 8-week-old mice (Figure 5A). Flow cytometric analyses of T cells isolated from lacrimal glands or lacrimal gland-draining cervical lymph nodes of nine-week-old WT NOD mice demonstrated enrichment of CXCR3-expressing T cells within lacrimal glands (Figure 5B). These differences were evident to varying degrees for each CD8^+^ T cells, CD4^+^ effector T cells, and CD4^+^ regulatory T cells (Figure 5C). When comparing the distribution of these T cell populations among the CXCR3^+^ T cells in glands or lymph nodes, we found that while CD8^+^ T cells were the dominant T cells expressing CXCR3 in the lymph nodes, the CXCR3-expressing gland-infiltrating T cells were dominated by Foxp3^+^CD4^+^ regulatory T cells (Figure 5D,E). 

## 3. Discussion

Type I IFNs have repeatedly been implicated in the pathogenesis of systemic autoimmune diseases such as SD [1,2,3,4]. However, the downstream consequences of such IFN signaling have not been clearly defined for each disease. We had previously identified a requisite role for type I IFN in lacrimal gland inflammation in NOD mice [19], and a pathogenic role for type I IFN in lacrimal and salivary gland inflammation was reported in another mouse model of SD [26]. Here, we identified potentially disease-relevant genes upregulated in lacrimal glands of NOD mice in a type I IFN-dependent manner to further define the downstream consequences of type I IFN signaling in the context of spontaneous lacrimal gland inflammation in the NOD mouse model of SD.

The most highly DE gene overexpressed in WT lacrimal glands in a type I IFN-dependent manner was *Oas2*, which is a member of the oligoadenylate synthase (OAS) family of enzymes that work in conjunction with RNase L to degrade viral RNA in infected cells [27]. This process results in cell death as a means to prevent viral survival and spread. While the association of OAS family members with autoimmunity is not new [27,28,29,30,31], the specific mechanisms by which OAS family members may contribute to autoimmunity is not well-described. In an individual with defects in clearing apoptotic cells, an anti-viral response via OAS family members could conceivably provide increased antigen or ligand for innate immune receptors such as Toll-like receptor 7 (TLR7) or other pattern recognition receptors. Alternatively, perhaps some dysregulation of the OAS family of proteins may lead to aberrant responses targeting self-RNA in the absence of an infectious trigger. Regardless, the associations of OAS members with autoimmunity encompasses multiple autoimmune diseases. OAS members were upregulated in muscle biopsies from children with juvenile dermatomyositis [28]. *OAS2* was among a limited set of genes upregulated in T cells of individuals with systemic lupus erythematosus (SLE), and this limited set of genes was able to reliably differentiate SLE from healthy controls and was associated with disease activity [32]. In SD, among genes with differential methylation patterns in labial minor salivary gland biopsy specimens, *OAS2* was found to have the strongest association with disease [33]. Genetic variant of another OAS family member, *OAS1*, was associated with SD and the variant resulted in isoforms purported to dysregulate type I IFN signaling [34]. The NOD mouse model of spontaneous lacrimal gland autoimmunity may help to elucidate the role of the OAS family in autoimmunity to determine whether their upregulation represents a pathogenic role versus a consequence of a dysregulated system that drives the aberrant immune response.

Among the most highly DE genes and the highest DE cytokine gene was *Il21*, which encodes IL-21, a pleiotropic cytokine with varied roles in innate and adaptive immunity and implicated in autoimmunity [22,35]. Specifically in SD, IL-21 was elevated in both serum [36,37] and labial minor salivary gland biopsy specimens of individuals with SD compared to controls [37] and in saliva of children with SD compared to controls [38]. A role for IL-21 in ocular inflammation in SD was demonstrated by elevation of IL-21 in tears and increased expression of *IL21* in conjunctival epithelial cells of individuals with SD compared to controls [39]. Moreover, the elevated tear IL-21 correlated with measures of increased ocular surface inflammation or tear dysfunction suggesting a pathogenic role. *Il21* expression was also elevated in cornea and conjunctiva in the CD25 KO mouse model of SD [40]. In NOD mice, *Il21* was detected in salivary glands and suppression with shRNA led to some improvement in salivary gland inflammation and function [41]. In lacrimal glands of WT NOD mice, IL-21-producing CD4^+^ T cells have been identified through single cell studies of gland-infiltrating T cells [42]. Phenotypic analyses suggested these IL-21-producing CD4^+^ T cells expressed some T follicular helper (Tfh) cell markers such as ICOS and PD1 but they did not express *Cxcr5* or *Bcl6*. Notably, they also expressed *Sostdc1*, which we found upregulated nearly 80-fold in WT lacrimal glands in a type I IFN-dependent manner. Whether the *Il21* upregulation in our WT NOD mouse lacrimal glands represents these Tfh-like cells, true Tfh cells, or another IL-21-producing population remains to be determined. Of note, though, *Cxcr5*, *Bcl6*, *Icos* and *Pdcd1* were upregulated in our WT lacrimal glands (5–23-fold) (Supplemental Table S1), and pathogenic contributions of Tfh cells in SD have been implicated in humans and animal models [43]. B cell hyperactivity is a hallmark in many SD patients, and the role of Tfh cells in driving B cell immune responses makes Tfh–B cell interactions a likely relevant immune axis in SD pathogenesis. However, the role of B cells in early SD is less clear given that B cell-deficient NOD mice are not completely protected from development of exocrine gland inflammation [44]. Early gland infiltrates are dominated by T cells, but B cells increase within the glands over time [42,45] suggesting the role of B cells may be greater later in disease. This may be especially relevant for the subset of SD patients who develop ectopic germinal centers (GC) within salivary glands providing a niche for chronic autoreactive B cell stimulation that increases risk for development of lymphoma. Recent transcriptomic analyses of salivary gland biopsy specimens from SD patients identified Tfh gene signature and increased *IL21* in specimens with ectopic GC and increased IL-21-producing Tfh in those with mucosa-associated lymphoid tissue lymphomas [46]. Our findings that *Il21* KO NOD mice are protected from WT-levels of lacrimal gland inflammation suggest a potential earlier role for IL-21 in development of lacrimal gland inflammation, but we did not detect significant upregulation of *Il21* in lacrimal glands at the earlier time point suggesting that IL-21 was not the only downstream signal mediating early disease development in an IFNAR1-dependent manner. This is not surprising given the many genes upregulated in lacrimal glands in an IFNAR1-dependent manner. Given the multiple cellular sources of IL-21, additional studies are needed to more directly assess the contributions of Tfh and other immune cell subsets in the production of IL-21 within lacrimal glands of NOD mice.

Immune cell trafficking is directed largely by chemokines. Among chemokines upregulated in WT NOD mouse lacrimal glands in a type I IFN-dependent manner, the most highly DE was *Cxcl13*. CXCL13 plays a role in directing Tfh cells into B cell follicles in the process of GC formation, which is consistent with the likely role for Tfh in lacrimal gland inflammation in NOD mice. CXCL13 has been implicated in SD in numerous human and animal studies [47]. However, the role of CXCL13 may be more significant in later propagation of the inflammatory process as *Cxcl13* was upregulated at the later, but not earlier, time point by qPCR in our studies. Additional chemokines upregulated in a type I IFN-dependent manner included multiple chemokines that were upregulated even at the earlier eight-week time point. These include *Cxcl9* and *Ccl19*, which we had previously identified as upregulated in lacrimal glands of older NOD mice in a type I IFN-dependent manner [19], and other cytokines including *Cxcl10*, *Ccl20*, *Ccl12*, and *Xcl1*. XCL1 (lymphotactin) is produced by T cells (especially activated CD8^+^ T cells and Th1 CD4^+^ T cells), NK cells, and NKT cells to recruit XCR1-expressing cells with recent evidence suggesting a role in promoting cross-presenting dendritic cells to stimulate cytotoxic CD8^+^ T cells [48,49,50]. *Xcl1* expression in NOD mouse lacrimal glands was previously reported [51]. CCL12 (monocyte chemotactic protein 5, MCP-5) is produced by innate immune cells and binds CCR2 to recruit innate and adaptive immune cells. CCL12 has been implicated in animal models of SD. *Ccl12* was upregulated in salivary glands in NZB/W mice when salivary gland inflammation was accelerated by TLR3 agonist-treatment [52]. In lacrimal glands, *Ccl12* was detected, albeit at low levels, in the MRL model [53]. CCL20 (macrophage inflammatory protein 3α, MIP-3α) is produced by several innate and adaptive immune cells and binds to CCR6, which is largely expressed on lymphocytes. CCL20 produced by keratinocytes recruits Th17 cells contributing to inflammation in psoriasis [54] but may contribute to an immunosuppressive environment through recruitment of immunomodulatory immune cells in the tumor microenvironment [55]. Whether CCL20 plays an inflammatory or compensatory anti-inflammatory role in NOD lacrimal glands is not known. CCL19 (macrophage inflammatory protein 3, MIP-3) binds CCR7 and plays a key role in localization of lymphocytes to secondary lymphoid organs or to ectopic lymphoid structures such as GC within exocrine glands in SD, a role it may play along with CXCL13 [47]. We and others have recently discussed the role of CCL19 in SD [19,47]. 

CXCL9 (monokine induced by gamma-IFN, MIG) and CXCL10 (IFN-γ induced protein 10, IP-10) each bind CXCR3 and have been implicated in SD in multiple human and animal studies [47]. Here we have extended our previous findings of upregulation of *Cxcl9* in lacrimal glands of older mice in a type I IFN-dependent manner [19] to demonstrate upregulation of *Cxcl9* and *Cxcl10* in a type I IFN-dependent manner at an early stage of lacrimal gland inflammation. Moreover, we detected CXCR3-expressing T cell populations enriched within lacrimal glands of WT NOD mice compared to the lacrimal gland-draining cervical lymph nodes. While all gland-infiltrating T cell subsets have larger CXCR3^+^ populations compared to the same subsets within the organ-draining cervical lymph nodes, we found significantly different distributions in T cell subsets within the CXCR3^+^ populations with CD8^+^ T cells dominating the CXCR3^+^ T cells in the lymph nodes but CD4^+^Foxp3^+^ regulatory T cells dominating the CXCR3^+^ T cell population within lacrimal glands. CXCR3^+^ regulatory T cells were previously found enriched within pancreatic islets in NOD mice and expressed *Tbx21* (encodes T-BET), and these T-BET^+^CXCR3^+^ regulatory T cells were shown to limit the development and progression of T1D [56]. Specifically, while the proportion of pancreatic regulatory T cells was relatively unchanged in the absence of these CXCR3^+^ regulatory T cells, their absence resulted in accelerated progression of T1D demonstrating their importance in modulating autoimmunity. Of note, T cells infiltrating lacrimal glands of NOD mice produce IFN-γ and TNF suggesting a strong type 1 immune response [57]. T-BET drives expression of CXCR3 in regulatory T cells to optimize modulation of type 1 inflammation [58,59]. Together, these data suggest that the CXCR3^+^ regulatory T cells enriched within lacrimal glands may limit the immunopathology during the lacrimal gland autoimmune response in NOD mice. In accordance with this, adoptive transfer of cervical lymph node cells depleted of CD4^+^Foxp3^+^ regulatory T cells resulted in extensive inflammation with diffuse rather than focal infiltrates in NOD-SCID recipient mice (our unpublished observations). However, in both T1D and SD-like manifestations in NOD mice, autoimmunity develops spontaneously suggesting that while CXCR3^+^ regulatory T cells may limit immunopathology in affected organs, they are incapable of preventing disease development in the first place. Whether this regulatory T cell dysfunction is driven by type I IFN is under investigation. 

In summary, type I IFN-signaling promoted upregulation of cytokines and chemokines within lacrimal glands leading to dysregulation of normal immune tolerance mechanism through complex phenotypic changes in innate and adaptive immune cells. Further studies are needed to more completely define the downstream consequences of type I IFN signaling and to specifically identify the lymphocytes and innate immune cells directly affected by the type I IFN signals. Moreover, the upstream signals driving the type I IFN response are also not fully elucidated, though our recent findings of a large common set of genes similarly upregulated in lacrimal glands in a TLR7-dependent manner suggest that TLR7 is one of those upstream type I IFN-inducing signals [60]. Ultimately, understanding the specific molecular mechanisms of type I IFN in SD-like autoimmunity may provide targets for novel therapeutics or for diagnostic or prognostic testing in SD and, potentially, other type I IFN-associated systemic autoimmune diseases. 

## 4. Materials and Methods

### 4.1. Mice 

NOD/ShiLtJ (NOD) mice were purchased from The Jackson Laboratory (Bar Harbor, ME, USA). *Ifnar1*-mutant (IFNAR1 KO) NOD mice generated by CRISPR/Cas9 mutagenesis were recently described [19]. *Il21*-mutant (IL-21 KO) NOD mice were generated by ZFN-mediated mutagenesis as previously described [61] to target exon 1 of the *Il21* gene. Constructs of the ZFN pairs that specifically target *Il21* were designed, assembled, and validated by Sigma-Aldrich (St. Louis, MO, USA). mRNAs encoding ZFN pairs were prepared in injection buffer (1 mM Tris-Cl, 0.1 mM EDTA, pH 7.4) at a concentration between 5–10 ng/µL and injected into the pronucleus of fertilized NOD one-cell embryos at the Medical College of Wisconsin Transgenic Core. Injected embryos were transferred to pseudopregnant CD-1 females. At weaning, DNA was extracted from tail tissues and screened for ZFN-induced mutation. Successful targeting was identified by PCR-amplifying genomic DNA using forward (5′-AAGATTTCCAGGCTGCAATG-3′) and reverse (5′-TGACAAACATGGCCTTACCA-3′) primers, followed by Sanger sequencing of the PCR products. Identified founders were backcrossed to NOD mice for one generation, followed by intercrossing to fix the mutation to homozygosity. ZFN binding sequences, target site, and deleted nucleotides are shown in Figure 3 for the 3 lines of IL-21 KO NOD mice used in this study. For lacrimal gland studies, male mice were used at ages indicated in the text. For diabetes studies, female mice were monitored weekly and diabetes onset was determined by two consecutive positive readings of glycosuria (>250 mg/dL) on a Diastix urine glucose strip (Bayer Diagnostics, Whippany, NJ, USA). Mice were maintained in our facilities in accordance with the Institutional Animal Care and Use Committee Guidelines, and reported studies were approved by Institutional Animal Care and Use Committees at the University of Iowa (#9021655, approved 19 March 2019) and Medical College of Wisconsin (AUA1863, approved 23 April 2019).

### 4.2. Histology and Lacrimal Gland Inflammation Quantitation

Exorbital lacrimal glands were fixed in buffered formalin, processed, embedded in paraffin, sectioned, and stained with H&E. Inflammation was quantified by standard focus scoring [57]. Briefly, foci of inflammation (defined as aggregates with a minimum of 50 mononuclear cells) were counted in a blinded manner by standard light microscopy (10× objective). Sections were scanned using a PathScan Enabler IV (Meyer Instruments, Houston, TX, USA) to obtain a whole-section, low-resolution, digital images from which tissue section areas were measured using ImageJ software [62]. Inflammation was reported as focus score, which is equal to the number of inflammatory foci per 4 mm^2^ tissue area. Representative whole-section images in the figures were obtained with a PathScan Enabler 5 (Meyer Instruments).

### 4.3. RNA Sequencing of Whole Lacrimal Gland RNA and Bioinformatics Analyses

RNA was isolated from lacrimal glands using RNeasy Plus Mini Kit (Qiagen, Valencia, CA, USA) following the manufacturer’s protocol. RNA samples were submitted to the Iowa Institute of Human Genetics Genomics Core Facility, where the samples were barcoded. Barcoded samples were pooled and sequenced using an Illumina HiSeq 4000 (Illumina, Inc., San Diego, CA, USA). Reads were demultiplexed and converted from the native Illumina BCL format to fastq format using an in-house python wrapper to Illumina’s “bcl2fastq” conversion utility. FASTQ data were processed with “bcbio-nextgen” in “RNA-seq” mode running on the Argon HPC resource at the University of Iowa. “Bcbio-nextgen” is a best-practices python NGS analysis pipeline available at the open-source “bcbio-nextgen” project (https://github.com/chapmanb/bcbio-nextgen, accessed on 15 February 2021; version 1.0.8). Reads were aligned to mm10/GRCm38 (Ensembl GRCm38.p6 v94 and GENCODE M19) reference genome/transcriptome using the ultra-rapid “hisat2” aligner (ver 2.1.0) [63]. Concurrently, reads were also quantified against the transcriptome using the “salmon” aligner (ver 0.9.1) [64], yielding estimated counts and values in length-normalized TPM (transcripts per million). Transcript-level estimates were converted to gene-level counts using the “tximport” package from Bioconductor [63]. Read and alignment quality control was performed with qualimap and samtools operating on the hisat2 BAM alignments [65,66,67]; counts from hisat2 alignment were not used for downstream analysis. All samples passed quality control with ~70% of reads mapping, and ~70% of mapped reads mapping to exonic regions. Sequencing depth ranged from 48–70 M reads/sample. Inspection of the PCA plot and lacrimal gland focus scores led to dropping one outlier sample from the IFNAR1 KO sample group (resulting in *n* = 3), which clustered with WT and had WT-level inflammation. No samples were dropped from the WT control (*n* = 3) group. Gene-level counts from salmon quantitation were used for differential gene expression analysis with DESeq2 [68] as recommended in the DESeq2 vignette (https://bioconductor.org/packages/release/bioc/vignettes/DESeq2/inst/doc/DESeq2.htm, accessed on 15 February 2021). Prior to calculating DE genes, the counts table was filtered to exclude genes where at least two WT samples had a sum of counts less than 30. This was done to prevent extreme outlier zero counts (i.e., dropouts) within the WT replicates from creating very large fold-change artifacts. Additional samples from TLR7 KO NOD mice were recently reported in comparison to these same WT samples [60]. Data have been deposited in NCBI’s Gene Expression Omnibus [69] and are accessible through GEO Series accession number GSE161184 (https://www.ncbi.nlm.nih.gov/geo/query/acc.cgi?acc=GSE161184, accessed on 15 February 2021). DE gene expression data was analyzed using iPathwayGuide (Advaita Bioinformatics, https://www.advaitabio.com/ipathwayguide, accessed on 15 February 2021) to detect and predict significantly impacted pathways, biological processes, and molecular interactions. These analyses implement an “impact analysis” approach, which considers the direction and type of all signals on a pathway along with the position, role and type of each gene [70,71,72,73]. 

### 4.4. Quantitative RT-PCR

Exorbital lacrimal glands were stored in RNA*later* (Invitrogen, Waltham, MA, USA) at −80 °C. Tissue samples were lysed and homogenized using the lysis buffer provided in the RNeasy Plus Mini Kit (Qiagen) using a disposable micro homogenizer (USA Scientific, Inc., Ocala, FL, USA). Total RNA extraction was performed using the RNeasy Plus Mini Kit according to manufacturer instructions. The SuperScript II Reverse Transcriptase Kit (Invitrogen) and random primers (Invitrogen) were used to generate cDNA. PCR was performed on a QS-7 FLEX Real Time PCR Systems (Applied Biosystems, Foster City, CA, USA) using Power SYBR Green PCR Master Mix (Applied Biosystems) and gene-specific primers (Table 6). The copy number (number of transcripts) of amplified product was calculated from a standard curve obtained by plotting known input concentrations of plasmid DNA. Expression levels of genes were normalized to *Gapdh*. 

### 4.5. IL-21 ELISA

Splenocytes were isolated and stimulated in vitro with anti-CD3 and anti-CD28 (1 g/mL each) (BD Biosciences, San Jose, CA, USA) in the presence of 100 ng/mL recombinant IL-6 (R&D Systems, Inc., Minneapolis, MN, USA) for 3 days. Tissue culture media were collected and analyzed for IL-21 by ELISA (eBioscience, San Diego, CA, USA) per manufacturer protocol in triplicate. 

### 4.6. Cell Isolation and Flow Cytometry

Cells were isolated from cervical LNs as previously described [56]. For lacrimal gland infiltrating cell isolation, glands were needle-diced then enzymatically dissociated by 125 U/mL collagenase type XI (Sigma-Aldrich, Saint Louis, MO, USA) in RPMI (ThermoFisher Scientific, Waltham, MA, USA) supplemented with 125 U/mL DNase I type II (Sigma-Aldrich) at 30 °C for 10 min on a rotator. Tissue was filtered through 70 μm nylon mesh strainer (ThermoFisher) then incubated at 30 °C for another 10 min in the same collagenase/DNase/RPMI before adding 0.5 M EDTA and incubating at room temperature for 10 min. Cells were pelleted by centrifugation at 233× *g* for 5 min, supernatant discarded, and red blood cells lysed by resuspending cells in ACK lysis buffer (Lonza, Mapleton, IL, USA) at room temperature for 5 min to obtain red blood cell-lysed single cell suspensions that were then prepared for flow cytometry. For discrimination of live/dead cells, fixable viability dye eFluor450 was used (eBiosciences). Cell surface staining was performed with the following antibodies: CD19-eFluor450 (clone eBio1D3, eBioscience), TCR-APC-eFluor780 (clone H57-597, eBioscience), CD4-APC (clone RM4-5, eBioscience), CD8-BV650 (clone 53-6.7, BD Biosciences), CXCR3-PE-Cy7 (clone CXCR3-173, BioLegend, San Diego, CA, USA). Intracellular staining for Foxp3-FITC (FJK-16s, eBioscience) was performed with the Foxp3 staining kit per manufacturer protocol. Flow cytometry data were acquired using a BD LSR II cytometer (BD Biosciences) and analyzed with FlowJo software (Treestar Inc, Ashland, OR, USA). For analyses, samples were gated based on size (FSC-A) and complexity (SSC-A) then for singlets (FSC-A by FSC-W) then for live T cells (TCR^+^CD19^−^). Of note, CD19 was conjugated to the same fluorophore as the live/dead to exclude dead and B cells in a single channel. Live singlet T cells were then further gated as indicated in figure legends and as depicted in representative gating plots (Supplemental Figure S1). 

### 4.7. Statistical Analyses 

Statistical analyses were performed with Prism 9.0.1 (GraphPad, Sand Diego, CA, USA) except for bioinformatics analyses using iPathwayGuide described above. Two-group comparisons of non-normally distributed data (focus scores, qPCR data) were performed with Mann-Whitney test. Comparisons of diabetes incidence were performed by Log-rank test. Two-group comparisons of data approximating normal distribution (flow cytometry data) were performed by paired t-test with data paired between different organs from the same individual mouse. All tests were two-tailed. *p* < 0.05 was considered significant.

## Figures and Tables

**Table 1:** Top 20 DE genes upregulated in lacrimal glands of WT mice compared to IFNAR1 KO mice ^1^.

Protein Name	Gene Symbol	Ensbl #	LogFC	*p*-Value ^2^
2′-5′-oligoadenylate synthase 2	*Oas2*	ENSMUSG00000032690	−8.42264	3.28 × 10^−^^8^
Titin	*Ttn*	ENSMUSG00000051747	−8.25179	0.034846
Troponin I, fast skeletal muscle	*Tnni2*	ENSMUSG00000031097	−6.8812	0.013172
Interleukin-21	*Il21*	ENSMUSG00000027718	−6.62862	0.000469
Hematopoietic SH2 domain-containing protein	*Hsh2d*	ENSMUSG00000062007	−6.54811	0.000154
Fc receptor-like protein 5	*Fcrl5*	ENSMUSG00000048031	−6.36972	0.000105
Sclerostin domain-containing protein 1	*Sostdc1*	ENSMUSG00000036169	−6.31561	0.000133
Alpha-actinin-3	*Actn3*	ENSMUSG00000006457	−6.27424	0.013255
PHD finger protein 11A	*Phf11a*	ENSMUSG00000044703	−6.10236	6.4 × 10^−5^
Sodium channel protein type 4 subunit alpha	*Scn4a*	ENSMUSG00000001027	−5.84383	8 × 10^−5^
Histidine-rich calcium-binding protein	*Hrc*	ENSMUSG00000038239	−5.82295	0.042974
Nebulin-related-anchoring protein	*Nrap*	ENSMUSG00000049134	−5.79493	0.034054
Sarcalumenin	*Srl*	ENSMUSG00000022519	−5.53226	0.005637
Complement component 4A	*C4a*	ENSMUSG00000015451	−5.49185	7.12 × 10^−6^
Interferon-activated gene 214	*Ifi214*	ENSMUSG00000070501	−5.45164	9.81 × 10^−5^
Grb2-binding adaptor protein, transmembrane	*Gapt*	ENSMUSG00000046006	−5.15291	0.000545
Glycogen phosphorylase, muscle form	*Pygm*	ENSMUSG00000032648	−5.11425	0.014735
Apolipoprotein L 10B	*Apol10b*	ENSMUSG00000050014	−5.10359	0.000322
Hepatitis A virus cellular receptor 1	*Havcr1*	ENSMUSG00000040405	−4.97945	0.001498
Alpha-1-antitrypsin 1-1	*Serpina1a*	ENSMUSG00000066366	−4.95592	0.020431

^1^ DE, differentially expressed; FC, fold-change (KO relative to WT); KO, knockout; WT, wild-type. ^2^ *p*-value adjusted for multiple comparisons by Benjamini-Hochberg procedure.

**Table 2:** Top 20 biological processes enriched in WT lacrimal glands ^1^.

Biological Process	DE Genes/Total	*p*-Value ^2^
immune system process	972/1850	1.00 × 10^−24^
immune response	609/1090	1.00 × 10^−24^
regulation of immune system process	577/1044	1.00 × 10^−24^
defense response	574/1042	1.00 × 10^−24^
response to external stimulus	840/1701	1.00 × 10^−24^
response to external biotic stimulus	512/943	1.00 × 10^−24^
response to other organism	512/943	1.00 × 10^−24^
response to biotic stimulus	520/967	1.00 × 10^−24^
cell activation	457/823	1.00 × 10^−24^
leukocyte activation	419/749	1.00 × 10^−24^
defense response to other organism	406/724	1.00 × 10^−24^
T cell activation	251/391	1.00 × 10^−24^
interspecies interaction between organisms	574/1120	1.00 × 10^−24^
positive regulation of immune system process	403/723	1.00 × 10^−24^
regulation of immune response	337/587	1.00 × 10^−24^
cytokine production	329/576	1.00 × 10^−24^
response to chemical	1107/2494	1.00 × 10^−24^
response to stimulus	2087/5175	1.00 × 10^−24^
lymphocyte activation	357/642	1.82 × 10^−24^
inflammatory response	270/454	1.02 × 10^−23^

^1^ DE, differentially expressed, WT, wild-type. ^2^ *p*-value with Bonferroni correction for multiple comparisons.

**Table 3:** Top 10 pathways overrepresented in WT lacrimal glands ^1^.

Pathway	DE Genes/Total	*p*-Value ^2^
Cytokine-cytokine receptor interaction	100/140	3.72 × 10^−6^
Viral protein interaction with cytokine and cytokine receptor	41/52	3.72 × 10^−6^
Complement and coagulation cascades	33/47	3.72 × 10^−6^
Natural killer cell mediated cytotoxicity	50/77	3.72 × 10^−6^
Chemokine signaling pathway	80/146	3.72 × 10^−6^
Antigen processing and presentation	47/64	3.72 × 10^−6^
Th17 cell differentiation	54/90	3.72 × 10^−6^
Staphylococcus aureus infection	36/42	3.72 × 10^−6^
Human T-cell leukemia virus 1 infection	110/201	1.06 × 10^−5^
Systemic lupus erythematosus	32/41	1.06 × 10^−5^

^1^ DE, differentially expressed, WT, wild-type. ^2^ *p*-value with Bonferroni correction for multiple comparisons.

**Table 4:** Top 20 DE genes in cytokine-cytokine receptor interaction pathway ^1^.

Gene	Ensbl #	LogFC	*p*-Value ^2^
*Il21*	ENSMUSG00000027718	−6.62862	0.000469
*Cxcr5*	ENSMUSG00000047880	−4.53441	0.000314
*Tnfrsf13c*	ENSMUSG00000068105	−4.41095	0.000366
*Ltb*	ENSMUSG00000024399	−4.11451	1.50 × 10^−5^
*Cxcl13*	ENSMUSG00000023078	−4.05289	0.000001
*Cxcl10*	ENSMUSG00000034855	−3.87295	2.75 × 10^−5^
*Ccl19*	ENSMUSG00000071005	−3.81592	0.005479
*Clcf1*	ENSMUSG00000040663	−3.69664	0.000425
*Lta*	ENSMUSG00000024402	−3.67207	0.010291
*Cxcl9*	ENSMUSG00000029417	−3.66073	0.014535
*Ccl20*	ENSMUSG00000026166	−3.6051	0.000001
*Cd40*	ENSMUSG00000017652	−3.59482	8.90 × 10^−5^
*Il21r*	ENSMUSG00000030745	−3.54375	2.11 × 10^−5^
*Ccr6*	ENSMUSG00000040899	−3.42886	3.37 × 10^−5^
*Cxcr4*	ENSMUSG00000045382	−3.36637	0.000001
*Gdf11*	ENSMUSG00000025352	−3.36559	0.000338
*Tnfsf8*	ENSMUSG00000028362	−3.32673	0.000137
*Ccl12*	ENSMUSG00000035352	−3.20344	0.000939
*Tnfrsf4*	ENSMUSG00000029075	−3.18646	0.001639
*Il2rg*	ENSMUSG00000031304	−3.12372	0.000157

^1^ DE, differentially expressed; FC, fold-change (KO relative to WT). ^2^ *p*-value with Bonferroni correction for multiple comparisons.

**Table 5:** Top 20 DE genes in viral protein interactions with cytokine and cytokine receptor pathway ^1^.

Gene	Ensbl #	LogFC	*p*-Value ^2^
*Cxcr5*	ENSMUSG00000047880	−4.53441	0.000314
*Cxcl13*	ENSMUSG00000023078	−4.05289	0.000001
*Cxcl10*	ENSMUSG00000034855	−3.87295	2.75 × 10^−5^
*Ccl19*	ENSMUSG00000071005	−3.81592	0.005479
*Lta*	ENSMUSG00000024402	−3.67207	0.010291
*Cxcl9*	ENSMUSG00000029417	−3.66073	0.014535
*Ccl20*	ENSMUSG00000026166	−3.6051	0.000001
*Ccr6*	ENSMUSG00000040899	−3.42886	3.37 × 10^−5^
*Cxcr4*	ENSMUSG00000045382	−3.36637	0.000001
*Ccl12*	ENSMUSG00000035352	−3.20344	0.000939
*Il2rg*	ENSMUSG00000031304	−3.12372	0.000157
*Ccr8*	ENSMUSG00000042262	−3.09507	0.044433
*Ccr1*	ENSMUSG00000025804	−3.07062	1.99 × 10^−6^
*Ccr7*	ENSMUSG00000037944	−3.04983	0.000386
*Il10ra*	ENSMUSG00000032089	−3.03792	3.455 × 10^−6^
*Cxcr3*	ENSMUSG00000050232	−2.87123	0.001774
*Ccl5*	ENSMUSG00000035042	−2.75027	1.000 × 10^−6^
*Xcl1*	ENSMUSG00000026573	−2.7486	0.014
*Il2rb*	ENSMUSG00000068227	−2.50687	0.002
*Ccr2*	ENSMUSG00000049103	−2.32380	1.388 × 10^−4^

^1^ DE, differentially expressed; FC, fold-change (KO relative to WT). ^2^ *p*-value with Bonferroni correction for multiple comparisons.

**Table 6:** Primer sequences for qPCR analyses.

Target	Sequence	References
*Il21* Fwd	GGACAGTGGCCCATAAATCA	[74]
*Il21* Rev	CAGGGTTTGATGGCTTGAGT	
*Oas2* Fwd	AAGAAGCGAAGGAGTGGCTG	
*Oas2* Rev	TGCCACAAGATCCCTCCTGTA	
*Cxcl13* Fwd	CATAGATCGGATTCAAGTTACGCC	[75]
*Cxcl13* Rev	TCTTGGTCCAGATCACAACTTCA	
*Cxcl10* Fwd	CATCCTGCTGGGTCTGAGTG	
*Cxcl10* Rev	ATTCTCACTGGCCCGTCATC	
*Cxcl9* Fwd	CCGAGGCACGATCCACTACA	[19]
*Cxcl9* Rev	CGAGTCCGGATCTAGGCAGGT	
*Ccl19* Fwd	ATGTGAATCACTCTGGCCCAGGAA	[19]
*Ccl19* Rev	AAGCGGCTTTATTGGAAGCTCTGC	
*Ccl20* Fwd	TTTTGGGATGGAATTGGACAC	[76]
*Ccl20* Rev	TGCAGGTGAAGCCTTCAACC	
*Ccl12* Fwd	GCTACAGGAGAATCACAAGCAGC	
*Ccl12* Rev	ACGTCTTATCCAAGTGGTTTATGG	
*Xcl1* Fwd	TTTGTCACCAAACGAGGACTAAA	
*Xcl1* Rev	CCAGTCAGGGTTATCGCTGTG	
*Gapdh* Fwd	TGTGTCCGTCGTGGATCT	
*Gapdh* Rev	CCTGCTTCACCACCTTCTTGA	

## Data Availability

RNA sequencing data are accessible through GEO Series accession number GSE161184 (https://www.ncbi.nlm.nih.gov/geo/query/acc.cgi?acc=GSE161184).

## Supplementary Materials

Supplementary materials can be found at https://www.mdpi.com/article/10.3390/ijms22073767/s1. Supplemental Table S1. Full DE gene list represented by Figure 2B volcano plot. Supplemental Table S2. Full list of enriched biological processes. Supplemental Table S3. Full list of enriched pathways. Supplemental Table S4. Full list of DE genes in cytokine-cytokine receptor pathway. Supplemental Table S5. Full list of DE genes in viral protein interactions with cytokine and cytokine receptor pathway. Supplemental Figure S1. Representative flow cytometry plots depicting the gating strategy.

## Abbreviations

Cerv LN	Cervical lymph node
DE	Differentially expressed
FC	Fold-change
H&E	Hematoxylin and eosin
IFN	Interferon
IFNAR	IFN alpha receptor
IL	Interleukin
KO	Knockout
NOD	Nonobese diabetic
OAS	Oligoadenylate synthase
SD	Sjögren’s disease
Tfh	T follicular helper
TLR	Toll-like receptor
T1D	Type 1 diabetes
WT	Wild-type
ZFN	Zinc-finger nuclease
