# Supplementary material for: Characterization of Type I Interferon-Associated Chemokines and Cytokines in Lacrimal Glands of Nonobese Diabetic Mice
Source: Int J Mol Sci. 2021 Apr 5;22(7):3767. doi: 10.3390/ijms22073767 (PMC8038628; doi:10.3390/ijms22073767)
Supplement: Supplementary file 1 [file ijms-22-03767-s001.zip › Supplemental Figure S1.docx]

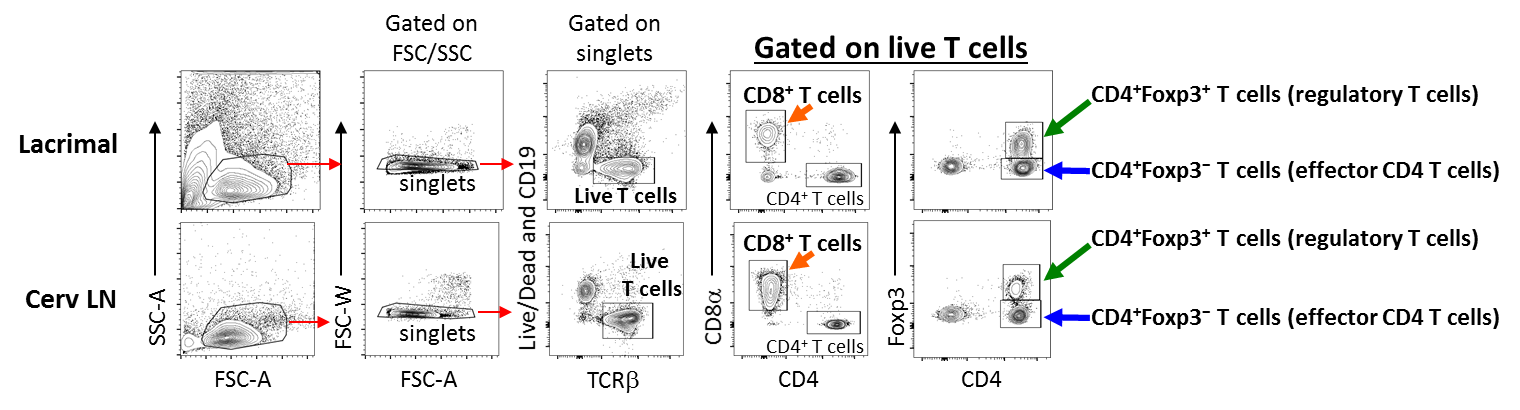


Supplemental Figure S1. Representative flow cytometry contour plots demonstrating the gating strategies used in our flow cytometry studies. Cells isolated from lacrimal glands (top) or cervical lymph nodes (cerv LN) (bottom) were isolated and stained for flow cytometric analyses. Red arrows indicate populations gated on for subsequent plot except the two columns on the right are both gated on live T cells.
